# Anti-platelet-activating factor, antibacterial, and antiradical activities of lipids extract from silver carp brain

**DOI:** 10.1186/1476-511X-12-94

**Published:** 2013-06-27

**Authors:** Caixia Wang, Wenshui Xia, Yanshun Xu, Qixing Jiang, Shanshan Yin, Yuwei Yang, Peipie Yu

**Affiliations:** 1 State Key Laboratory of Food Science and Technology, School of Food Science and Technology, Jiangnan University, Wuxi 214122, China

**Keywords:** Silver carp, Brain lipids, Platelet-activating factor (PAF), PAF antagonists, Antibacterial, Antiradical

## Abstract

**Background:**

Epidemiological studies have verified the protective role of fish lipids in cardiovascular diseases. However, the effects of fish lipids on health boost remain undefined. Large amounts of by-products, such as fish brain which contains high level of lipids, are produced with silver carp processing. Fish brain is rich in bioactive lipids which are overwhelmingly effective in preventing cardiovascular diseases. The aim of this study was to elucidate the pharmacological activities of silver carp brain lipids against diseases by inhibiting platelet-activating factor (PAF), suppressing bacterial growth and scavenging free radicals.

**Methods:**

Total lipids (TL) were extracted from silver carp brain and separated into polar lipids (PL) and neutral lipids (NL). The capabilities of the lipid fractions in aggregating washed rabbit platelet or in inhibiting PAF-induced platelet aggregation were tested. Their antibacterial and antiradical activities were studied as well.

**Results:**

The lipid fractions exhibited strong inhibitory activities, and the activity of TL was mainly attributed to NL. TL exhibited antibacterial activity towards *Staphylococcus aureus*, while NL managed to fight against *S. aureus* and *Escherichia coli*. PL excelled TL and NL in simultaneously suppressing the growths of *Shigella dysenteriae* and *Salmonella typhi* besides those of *S. aureus* and *E. coli.* The scavenging effect of PL on 2,2-diphenyl-1-picrylhydrazyl radical was considerably higher than those of TL and NL.

**Conclusion:**

The present study may help to explain the protective role of fish lipids against diseases and may be responsible for the effectiveness of fish brain in benefiting health.

## Introduction

Fish lipids have aroused intensive clinical interest due to the pharmacological activities against various diseases, the consumption of which prevents human chronic inflammatory, cardiovascular diseases and cancer [1]. Although studies have been devoted to analyzing and explaining the role of fish lipids against various degenerative diseases, further studies are still needed and the types of fishes involved remain limited.

Silver carp (*Hypophthalmichthys molitrix*), as one of the main freshwater fish species in China, contributes 3,713,922 tons in 2011 [2]. However, over half of total fishery products are being discarded as inedible by-products every year [3]. Traditionally considered physically and intelligently nutritious and beneficial, fish brain, as a derivative though, is popular in China and is thus of industrial interest nowadays. The advantages of fish brain stem from the high content and quality of lipids [4] which, however, has seldom been accessed regarding health issues.

Platelet-activating factor (PAF, 1-O-alkyl-2-acetyl-sn-glyceryl-3-phosphocholine), which is key in atherosclerosis development, is also an extremely potent aggregating agent participating in the inflammatory development of plaque and the blockage of blood vessels that finally lead to coronary heart disease [5]. Many foodstuffs contain molecules that inhibit the activity of PAF as PAF antagonists that are nutritionally valuable because they prevent platelets aggregation in arteries and atheromatosis generation simultaneously. Previous researches have revealed that lipids extracted from wild haddock and coley exhibited PAF-inhibiting activities [6].

Many foods primarily deteriorate because of microbes that give rise to the loss of quality and safety, on which people worldwide are concerning more because foodborne diseases are subject to outbreak due to pathogenic and spoilage microorganisms in foods [7]. In addition, both Gram-positive and Gram-negative bacteria evoke a state of shock that is characterized by cardiovascular collapse [8]. Numerous bacteria are able to produce PAF [9]. For instance, PAF can be synthesized by being stimulated with Stap*hylococcus aureus* toxins or it can be released by *Escherichia coli*[10]. Given that PAF is an initial trigger in atherosclerosis [11], foodstuffs, which exert antibacterial activities, suppress the production of PAF, and therefore play a positive role in preventing atherosclerosis. Previous studies have demonstrated the lipids extracted from red shrimp brain, marine sponge, sea bass and gilthead sea bream exerted antibacterial activities [10,12,13]. Besides, antimicrobial agents have been prepared from the lipids of marine fishes [14].

Free radicals are engaged in the development of cardiovascular disease, cancer, liver disease and other chronic and inflammatory diseases based on oxidative damages [15], the scavenging of which therefore has been spotlighted. It has previously been reported that smokers displayed enhanced antioxidant capacity after being administered with fish oil [16] which is associated with the reduced susceptibility of myocytes to injury-inducing reactive oxygen species upon dietary supplementation [17].

In the present study, the lipid fractions of silver carp brain were tested for their biological activities concerning platelet aggregation induction or PAF-induced platelet aggregation inhibition. The antibacterial activities of the lipids against *E. coli*, *S. aureus*, *Shigella dysenteriae*, *Salmonella typhi* and *Listeria monocytogenes* were examined. Moreover, their radical-scavenging effects were determined by utilizing 2,2-diphenyl-1-picrylhydrazyl (DPPH). The results may assist to explain the pharmacological activities of fish lipids against various diseases. Furthermore, the results may shed light on the commercial and industrial utilization of silver carp brain lipids as eligible bioactive ones.

## Material and methods

### Materials

Silver carps (1.5-2 kg) were purchased from a local market (Wuxi, Jiangsu Province) in April 2012. Live fish (n≥100) in water were transported to the laboratory, and then weighed and decapitated individually. The brain was removed, collected and homogenized. The prepared brain samples were kept at −70°C before lipids extraction.

### Isolation of lipid fractions

Lipids were extracted by the method described by Folch et al. [18]. Total lipids (TL) were separated into neutral lipids (NL) and polar lipids (PL) by counter-current distribution [19].

### Platelet aggregation assay

The biological activities of TL, NL and PL against washed rabbit platelets were tested as described previously [20], Briefly, PAF (Sigma) and the examined samples were dissolved in 2.5 mg bovine serum albumin (BSA) per ml of saline. Various concentrations of the sample were placed in an aggregometer (CHRONO-LOG, USA) cuvette and the resultant aggregatory effect was measured as the percentage of maximum reversible aggregation. The aggregatory activity of the sample was expressed as the amount inducing 50% of maximum reversible aggregation that was defined as EC_50_, i.e. equivalent concentration for 50% reversible aggregation. Samples at different concentrations were placed in an aggregometer cuvette to determine their abilities in inhibiting PAF-induced aggregation. The platelet aggregation induced by PAF (2.5×10^-11^ M, final concentration in the cuvette) was measured before (considered as 0% inhibition) and after adding the sample. Consequently, the dependence of percent inhibition on sample concentration was plotted, from which the concentration that inhibited 50% of PAF-induced aggregation was calculated and defined as IC_50_, i.e. concentration for 50% inhibition.

### Antibacterial assay

#### Antibacterial screening

The antibacterial activities of TL, NL and PL against *E. coli* O157:H7 NCTC 12079, *S. aureus* NCBF 1499, *L. monocytogenes* NCTC 10527, *S. dysenteriae* ATCC 51302, and *S. typhi* ATCC 5784 were analyzed. The bacterial strains were cultured in Mueller-Hinton Broth except *E. coli* was cultured in Nutrient Broth otherwise. The antimicrobial activities of the lipids were determined by a modified paper disc diffusion method [10]. Briefly, a suspension of the test microorganism (10^8^ CFU/ml) was spread on solid media plates that were then incubated at 4°C for 2 h. Sterile 6 mm diameter filter paper discs were impregnated with 15 μl of diluted lipids and dried under nitrogen stream. Then the sterile paper discs were placed on an agar Petri dish and incubated at 37°C for 24 h, and a paper disc impregnated with 15 μl of mixed chloroform and methanol (1:1) was used as the control. After incubation, all dishes were observed for the zones of inhibition and the corresponding disc diameters (DD) were measured in millimeters.

#### Determination of minimum inhibitory concentration (MIC) and minimum bactericidal concentration (MBC)

The MICs of TL, NL and PL against the bacterial strains were evaluated according to the method of broth dilution [21]. An aliquot of 5 ml nutrient broth supplemented with Tween 80 (0.5% v/v) was placed into each tube, and then all tubes were autoclaved at 121°C for 20 min. The lipids were added in the tubes and the final concentrations were kept at 0.1-25.6 mg/ml, to which was then added the test bacteria suspension that was kept at the final inoculum size of 10^6^ CFU/ml. Thereafter the inoculated tubes were incubated at 37°C for 24 h. Culture medium without bacteria suspension was prepared as the control. MIC is defined as the lowest concentration of the lipids at which the microorganism does not demonstrate visible growth. Microorganism growth was indicated by the turbidity.

To determine MBC, broth was taken from each tube and inoculated in agar Petri dishes for 24 h at 37°C. MBC is defined as the lowest concentration of the lipids at which the inoculated microorganism was completely killed.

### Antiradical assay

DPPH (Sigma) was dissolved in anhydrous ethanol at 0.1 mM. TL, NL and PL were dissolved in anhydrous ethanol as well at 0.05-51.2 mg/ml. Lipids solution (2 ml) was added to 2 ml of DPPH solution. The mixture was shaken vigorously and left for 30 min in dark at room temperature. The absorbance was then measured at 517 nm within 10 min [22]. The sample and DPPH solutions were replaced with 2 ml of anhydrous ethanol each as the control and blank respectively. The scavenging effect (d %) on DPPH radical was calculated according to the following equation:

$$\mathrm{d\%}=\frac{\left(A_{\mathrm{control}}-\left(A_{\mathrm{sample}}-A_{\mathrm{blank}}\right)\right)}{A_{\mathrm{control}}}100\%$$

The inhibition concentration at which 50% of the DPPH radicals were scavenged (IC_50_’) was obtained by plotting the scavenging activity against the sample concentration.

### Statistics

All analyses were performed in triplicate, and all data were described as mean ± standard deviation (SD). Significant differences were defined at p<0.05. Group means were compared using ANOVA and Duncan’s tests. All statistics were performed using SPSS statistical package (version 18.0).

## Results

### Platelet aggregation properties

The abilities of lipid fractions (TL, NL and PL) in aggregating washed rabbit platelets or in inhibiting PAF-induced platelet aggregation were tested. The biological activity of each lipid fraction and their corresponding EC_50_ or IC_50_ value are shown in Table 1. All fractions were highly inhibitory. As a mixture of NL and PL, the inhibitory activity of TL was mainly attributed to NL since NL showed significantly higher inhibitory activities than PL.

**Table 1:** Biological activities (aggregation of washed rabbit platelets or inhibition of PAF-induced platelet aggregation) of lipid fractions from silver carp brain

**Lipid fraction**	**Content ( g /50g brain tissue)**	**Action**	**EC**_**50 **_**(μg)**	**IC**_**50 **_**(μg)**
Total lipid	10.26±0.11^c^	Inhibition	–	104.13±21.33^b^
Neutral lipid	7.92±0.08^b^	Inhibition	–	2.35±0.66^a^
Polar lipid	2.34±0.07^a^	Inhibition	–	87.47±6.53^b^

Data were presented as means ± SD (n=3), and value in the same row with different superscript letters denotes significant difference (p<0.05).

EC_50_ expresses the amount of lipids which induces 50% of the maximum reversible aggregation; IC_50_ expresses the amount of lipids which inhibits 50% platelet inhibition against 2.5×10^-11^M PAF.

### Antibacterial activities

The antibacterial activities of TL, NL and PL from silver carp brain against five bacteria were investigated (Table 2). TL exhibited antibacterial activity towards *S. aureus*, while NL exhibited antibacterial activity towards *S. aureus* and *E. coli*, and PL showed antibacterial activity towards *S. dysenteriae* and *S. typhi* besides *S. aureus* and *E. coli*. The DD, MIC and MBC of TL, NL and PL towards *S. aureus* followed the descending order of PL>TL>NL, and the DD of PL towards *E. coli* was larger than that of NL, indicating that PL was of higher antibacterial activity than TL and NL.

**Table 2:** Zones of growth inhibition, MIC and MBC of lipid fractions from silver carp brain

**Strains tested**	**Total lipid**			**Neutral lipid**			**Polar lipid**		
	**DD**	**MIC**	**MBC**	**DD**	**MIC**	**MBC**	**DD**	**MIC**	**MBC**
	**mm**	**mg/ml**	**mg/ml**	**mm**	**mg/ml**	**mg/ml**	**mm**	**mg/ml**	**mg/ml**
*Escherichia coli*	NI	–	–	7.22±0.27	0.8	0.8	7.74±0.32	0.8	1.6
*Staphylococcus aureus*	10.48±0.93^a^	6.4	6.4	9.07±0.44^a^	12.8	12.8	12.57±0.75^b^	1.6	1.6
*Shigella dysenteriae*	NI	–	–	NI	–	–	7.48±0.30	0.8	1.6
*Salmonella typhi*	NI	–	–	NI	–	–	7.28±0.18	1.6	3.2
*Listeria monocytogenes*	NI	–	–	NI	–	–	NI	–	–

Data were presented as means ± SD (n=3), and value in the same line with different superscript letters denotes significant difference (p<0.05).

Tested at a concentration of 6.0 mg per disc.

*DD* disc diameters of the inhibition zones, including the diameter of the filter paper disc (6 mm), *MIC* minimum inhibitory concentration, *MBC* minimum bactericidal concentration, *NI* no inhibition.

### Antiradical activities

**Figure 1:**
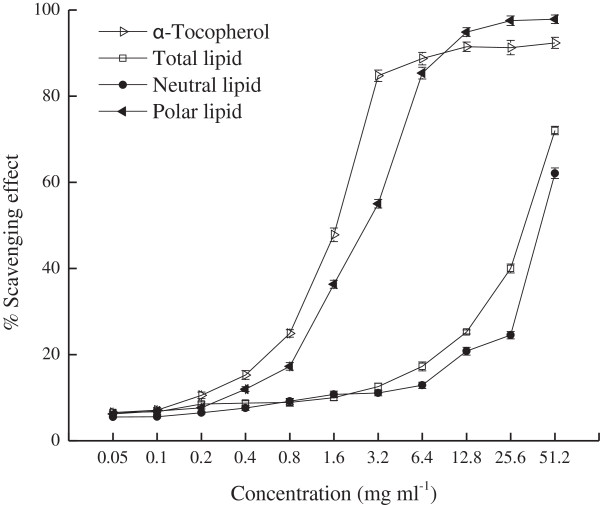
**DPPH radical-scavenging effects of α-tocopherol and lipid fractions from silver carp brain.** IC_50_’ (inhibition concentration at which 50% of the DPPH radicals were scavenged): α-Tocopherol, 1.76 mg/ml; Total Lipid, 35.02 mg/ml; Neutral Lipid, 44.16 mg/ml; Polar Lipid, 2.91 mg/ml.

DPPH molecule, which contains a stable free radical, has been widely used to evaluate radical scavenging abilities. The DPPH radical-scavenging effects of TL, NL and PL as well as α-tocopherol were determined (Figure 1). PL and α-tocopherol scavenged radicals significantly more potently than TL and NL did, with the IC_50_’ values of 2.91 mg/ml, 1.76 mg/ml, 35.02 mg/ml and 44.16 mg/ml, respectively. Meanwhile, α-tocopherol, at 0.4-6.4 mg/ml, scavenged radicals more effectively than PL did, with scavenging rates skyrocketing from approximately 10% to 85%. However, when exceeding 12.8 mg/ml, the scavenging effect of PL began to surpass that of α-tocopherol. Moreover, the scavenging effects of TL and NL were also augmented with elevating concentration, but they worked considerably inferior to PL. At 0.05-1.6 mg/ml, the scavenging effects of TL and NL were increased slightly from approximately 6% to 10%, after which the scavenging effect of TL was increased more rapidly than that of NL at equal concentrations.

## Discussion

ω3 polyunsaturated fatty acids (ω3 PUFAs) in fish oil are potentially cardioprotective and antithrombotic [23]. However, other substances, apart from ω3 PUFAs, may be responsible for the antithrombotic properties of fish oil [24]. PAF antagonists in foodstuffs are nutritionally valuable considering the importance of platelet activation and thrombosis in cardiovascular diseases as well as the pivotal role of PAF in atherogenesis. PAF antagonists have been isolated from wild and cultured specimens of sea bass and gilthead sea bream [25]. Previous studies have verified that the lipids extracted from cultured gilthead sea bream, wild gilthead sea bream, sea bass, and plaice had bimodal effects on washed rabbit platelet. However, the lipids from cultured rainbow trout and golden trout were only aggregatory, while those from wild haddock and coley were only inhibitory. Notably, the lipids from cultured sea bass were of aggregatory, inhibitory and bimodal effects in different analyses [6,10,25]. Therefore, the biological activities of fish lipids depend on species indeed. The aggregatory property and the PAF antagonistic activity of TL mainly originate from PL and NL respectively [6,25]. Lipid fractions are intrinsically a mixture of lipid molecules that are potentially aggregatory or inhibitory. The final activity is governed by the relative ability of each molecule to aggregate platelet or to inhibit PAF-induced platelet aggregation and by the relative amount of each molecule in the mixture. It is also worth mentioning that the compounds with PAF-like activity in lipids are also beneficial as they act as weak PAF agonists and compete with PAF for common binding sites during the formation of atheromatic plaque in blood arteries, thus protecting them from atheromatosis generation. In other words, the compounds are literally PAF inhibitors.

Fish lipids are sufficient in ω3 PUFAs which have been proven antibacterially active [26]. For instance, lipids of red shrimp brain inhibited the growths of *S. aureus*, *S. dysenteriae* and *S. typhi*[13], and TL from sea bass and gilthead sea bream inhibited the growth of *S. aureus*, while NL and PL suppressed those of *E. coli* and *S. aureus*[10]. Fatty acids (FAs), which fight against many microorganisms [27], usually function relying on long-chain unsaturated fatty acids (LCUFAs) including oleic acid, linoleic acid, and linolenic acid [28]. They all confront bacteria through several mechanisms of action, all of which primarily involve the perturbation of bacterial cell membrane [29]. The bactericidal activity of LCUFAs against *S. aureus* enhanced with increasing degree of unsaturation [12]. PL includes phospholipids which are richer in LCUFAs due to their functional role [30]. Our previous research has confirmed abundant LCUFAs in silver carp brain especially in PL, which may be responsible for the antibacterial activities of the lipid fractions. In addition, cardioprotective sphingolipids [31], exist more abundantly in fish brain than in muscle [32], in which sphingosine is highly antibacterial [33]. On the other hand, PAF antagonists also contribute to the antibacterial activities of lipid fractions by having antibacterial activities [10] and by inhibiting bacterial-induced inflammatory actions [8]. Consequently, the differences between the antibacterial activities of lipids from silver carp brain and those from other fish species can be ascribed to their different FA profiles, sphingolipids contents and PAF antagonist patterns.

Richard et al. reported that FA micelles scavenged superoxide in an unsaturation-dependent manner [34]. In this study, the strong DPPH radical-scavenging effect of PL mainly results from the higher content of PUFAs, especially ω3 PUFAs, in PL than those in TL and NL (previously proven by our group). It is worth noting that in vivo experiments have demonstrated reduced excretion of lipid peroxidation products after PUFAs intake [35], and in vitro data have shown that reactive oxygen species are not prone to formation after ω3 FAs supplements. Thus, ω3 FAs may indirectly act as anti- rather than pro-oxidant in vascular endothelial cells, hence diminishing inflammation and, in turn, the risks of atherosclerosis and cardiovascular disease [34].

## Conclusion

In summary, we herein have verified that the lipid fractions from silver carp brain were highly active PAF antagonists, and that NL was mainly responsible for the inhibitory actions of TL. All the lipid fractions were capable of battling bacteria, of which PL was most superb. In the meantime, PL showed higher antiradical activities than TL and NL. The anti-PAF, antibacterial and antiradical activities of the lipid fractions from silver carp brain are conducive to clarifying the protective roles of fish lipids against diseases and probably rendering fish brain efficacious in health boost.

## Competing interests

The authors declare that they have no competing interests.

## Authors’ contributions

WSX and CXW designed the study. CXW carried out the experiments, performed the statistical analysis and drafted the manuscript. YSX and QXJ helped to revise the manuscript. PPY contributed to the providing of essential materials. SSY and YWY helped to carry out the experiments. All authors read and approved the final manuscript.
